# Proton transport drives allosteric conformational changes in the MmpL3 transporter

**DOI:** 10.1371/journal.pcbi.1014880

**Published:** 2026-10-21

**Authors:** Lixinhao Yang, E. Jeffrey North, Helen I. Zgurskaya, Mary Jackson, James C. Gumbart

**Affiliations:** 1 School of Chemistry and Biochemistry, Georgia Institute of Technology, Atlanta, Georgia, United States of America; 2 Department of Pharmacy Sciences, Creighton University, Omaha, Nebraska, United States of America; 3 Department of Chemistry and Biochemistry, University of Oklahoma, Norman, Oklahoma, United States of America; 4 Mycobacteria Research Laboratories, Department of Microbiology, Immunology and Pathology, Colorado State University, Fort Collins, Colorado, United States of America; 5 School of Physics, Georgia Institute of Technology, Atlanta, Georgia, United States of America; Universitat des Saarlandes, GERMANY

## Abstract

Tuberculosis (TB), caused by *Mycobacterium tuberculosis* (*Mtb*), remains a global health crisis, further complicated by increasing drug resistance. The mycobacterial membrane protein MmpL3, which is essential for transporting cell wall precursors such as trehalose monomycolate (TMM), is a promising therapeutic target. However, the mechanistic link between proton-driven dynamics and substrate transport in MmpL3 is not fully understood. In this study, we use molecular dynamics simulations to investigate how protonation of conserved Asp-Tyr dyads (Asp256-Tyr646 and Asp645-Tyr257) in *Mycobacterium smegmatis* (*Msmg*) MmpL3 regulates conformational changes and TMM binding. Protonation at Asp256 induces an open dyad conformation, increases local water accessibility, and is associated with a tendency toward more open configurations of the periplasmic binding pocket. In contrast, protonation at Asp645 stabilizes a closed dyad conformation, reduces water accessibility, and narrows the transmembrane binding pocket. These findings support a proposed sequential protonation model in which protonation-dependent conformational changes are allosterically coupled to substrate translocation.

## Author summary

Tuberculosis remains one of the deadliest infectious diseases worldwide, in part because the bacterium builds an unusually strong cell wall that is difficult for drugs to penetrate. MmpL3 is essential for assembling this cell wall and is therefore an important target for new drugs. However, it has not been clear how this transporter uses protons to power lipid movement. In this work, we used molecular dynamics simulations to examine how protonation at two conserved sites controls the structure and behavior of MmpL3. We focused on two conserved sites inside the protein and found that protonation at each site produces different structural effects. One protonation state favors a more open and hydrated local environment, while the other favors a more closed and less hydrated configuration. These changes support a stepwise mechanism in which protonation acts as a molecular switch to coordinate transport. By clarifying how proton-driven conformational changes power MmpL3 function, our work provides insight into a critical process in mycobacterial cell wall construction and may help guide future efforts to develop new tuberculosis therapies.

## Introduction

Tuberculosis (TB), caused by *Mycobacterium tuberculosis (Mtb)*, remains a persistent global health threat. Each year, an estimated 10.7 million people are diagnosed with TB, and approximately 1.23 million individuals die from the disease [1], making it one of the leading causes of mortality among infectious diseases worldwide. Current treatment regimens are lengthy, often requiring six months or more of combination antibiotic therapy, which poses challenges for patient adherence and increases the risk of adverse side effects [2–5]. Moreover, the emergence and spread of multidrug-resistant (MDR) and extensively drug-resistant (XDR) strains have rendered many existing treatments less effective [6,7], further complicating global TB control efforts [8,9].

The unique and complex cell wall of mycobacteria presents a formidable challenge in the development of effective anti-TB drugs [10–13]. This robust barrier, enriched in mycolic acids and other lipids, markedly reduces permeability and limits the intracellular accumulation of many antibiotics, thereby contributing to intrinsic drug resistance of *Mtb* [14–17]. The mycobacterial cell envelope also plays a central role in bacterial physiology and virulence, supporting survival and replication within host cells [10,17–20]. The structural complexity and unusual composition of the envelope make it difficult to target with conventional antibiotics, highlighting the need for innovative approaches that can disrupt its biosynthesis or integrity.

One of the critical proteins required for mycobacterial survival and cell wall biogenesis is MmpL3 (mycobacterial membrane protein Large 3) [21–25], an essential inner membrane transporter required for mycolic acids, major cell wall constituents, to reach their extracytoplasmic location [22,26,27]. During the transport cycle, TMM is known to bind spontaneously within a transmembrane pocket of MmpL3 [28], is subsequently transferred to a periplasmic binding pocket [29], and is subsequently handed off to downstream periplasmic components. Recent studies have suggested that allosteric coupling exists between proton transport and the periplasmic pocket [30,31], implying that MmpL3 undergoes conformational rearrangements to facilitate TMM movement. As a highly conserved transporter across mycobacterial species [32], MmpL3 is indispensable for bacterial growth and has been recognized as a promising therapeutic target [33–37]. Inhibition of MmpL3 leads to impairment of cell wall biosynthesis and ultimately the death of the bacterium [26,27,34,36,38–40], underscoring its appeal for the development of new anti-TB agents.

Despite its critical role in mycobacterial physiology, the mechanistic details underlying MmpL3 function remain incompletely understood. MmpL3 is known to operate as a proton-coupled transporter, in which proton flow across the membrane drives the translocation of TMM [41]. Within the central transmembrane domain, two conserved Asp–Tyr dyads located on TM4 and TM10 have been proposed to mediate proton transfer during the transport cycle (Fig 1A) [26,42–44]. In the proposed transport cycle, protons are thought to move down the proton gradient, from the periplasmic side toward the cytoplasmic side. This proton movement provides the energy to drive TMM translocation in the opposite direction, from the transmembrane binding site toward the periplasmic pocket for subsequent handoff to cell-wall biosynthesis machinery. This proton-driven mechanism is central to MmpL3’s function, making it a potential target for disruption by small-molecule inhibitors. By binding to MmpL3, ligands can disrupt proton translocation or alter the conformational states of the protein, thereby halting its functional cycle [44–47]. Notably, MmpL3 is highly conserved across mycobacterial species [32,48,49]. In particular, *Mtb* MmpL3 can rescue the viability of a *Mycobacterium smegmatis* (*Msmg*) MmpL3 null mutant [26], supporting the use of *Msmg* MmpL3 as a model system for mechanistic studies. As a result, *Msmg* MmpL3 has been widely employed to investigate transporter structure, dynamics, and inhibitor development [22,44,49].

**Fig 1:**
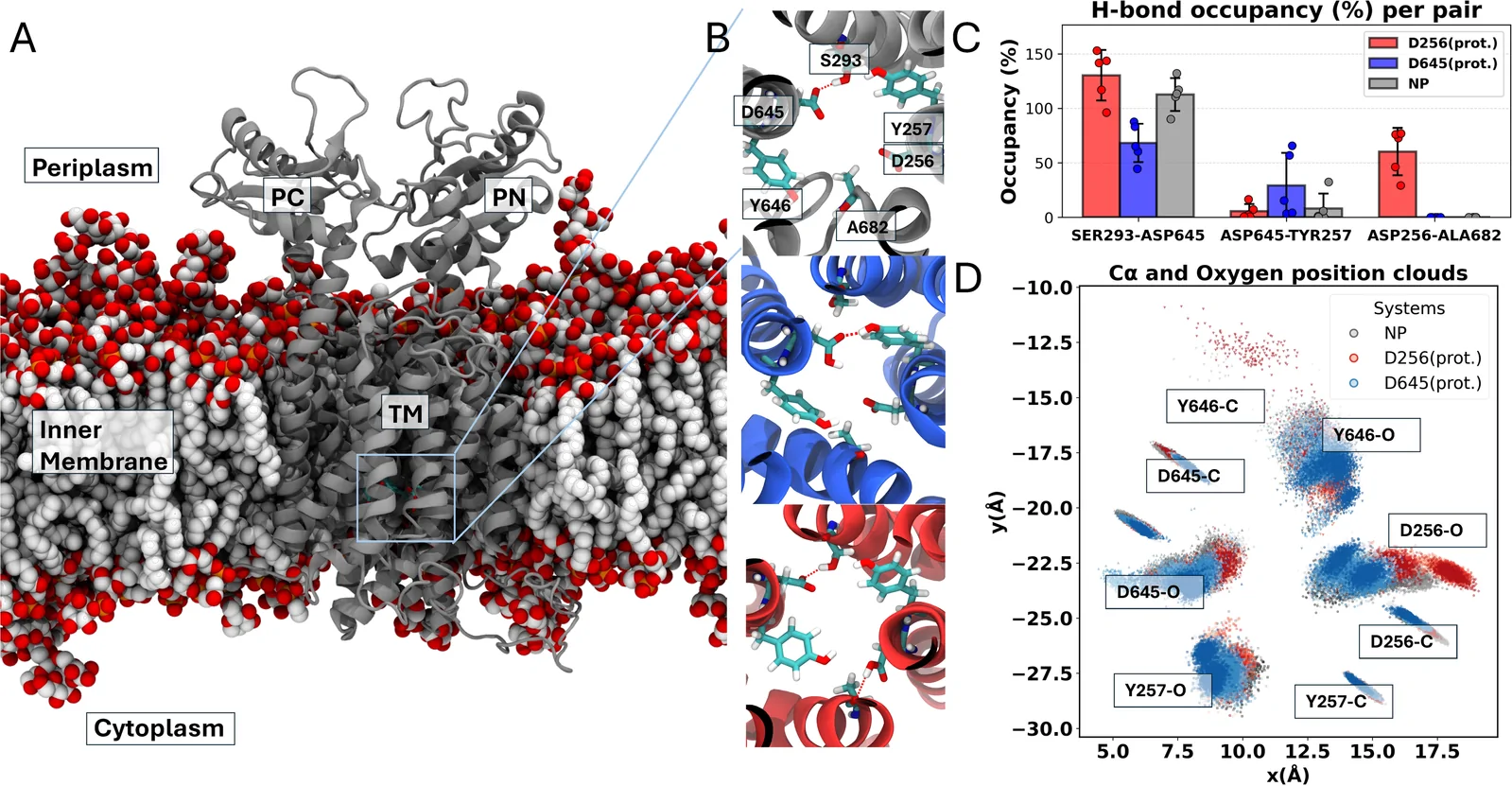
Protonation modulates Asp–Tyr dyad geometry and local interactions in MmpL3. **(A)** Overview of the *Msmg* unprotonated MmpL3 embedded in a native-like inner membrane, indicating the periplasmic (PN, PC) and transmembrane (TM) domains. **(B)** Representative local H-bond networks near the Asp-Tyr dyads in the NP, D256(prot.), and D645(prot.) states, viewed from the periplasmic side. The distinct interaction patterns illustrate how protonation alters the dyad-proximal H-bond network. **(C)** H-bond occupancies for key dyad-associated residue pairs Ser293–Asp645, Asp645–Tyr257, and Asp256–Ala682 in NP (grey), D256(prot.) (red), and D645(prot.) (blue); values represent mean ± SD over five replicas, with pairwise Welch’s *t*-test *p* values reported in S1 Fig. **(D)** Two-dimensional posi*t*ional distributions of Cα atoms and side-chain oxygen atoms for Asp–Tyr dyads, averaged over all replicas.

In this study, we used a comprehensive suite of molecular dynamics (MD) simulations to investigate how protonation of the dyads influences structure and function in *Msmg*. By embedding MmpL3 in a native-like mycobacterial inner membrane, we mapped conformational shifts and identified changes in water accessibility near the Asp-Tyr dyads. To further elucidate the role of proton-driven transport, we analyzed the impact of protonation on the periplasmic domain and TMM binding at both the transmembrane and periplasmic pockets. Together, these simulations provide a mechanistic perspective on how protonation events allosterically regulate MmpL3’s transport cycle, paving the way for future structure-based efforts to develop potent anti-TB agents.

## Materials and methods

### Model building

For *Msmg* MmpL3, we utilized a crystal structure (PDB: 6AJG) as initial coordinates of the unprotonated system [44]. The resolution of the selected structure is 2.6 Å. The pKa values of all titratable residues were predicted using the PROPKA web server [50], and their protonation states were assigned accordingly. His101 was modeled as doubly protonated (HSP), whereas the remaining histidines were modeled as neutral HSD tautomers; Cys24 and Cys735 were kept as free thiols, and the N and C termini were neutralized with acetyl (ACE) and amide (CT2) groups, respectively. For the protonated systems, Asp256 or Asp645 was selectively modeled as a neutral, singly protonated carboxylic acid using the CHARMM ASPP patch via the Psfgen plugin in VMD [51], while both residues remained deprotonated in the NP state. TMM coordinates were extracted from a crystal structure (PDB: 7N6B) [29] and aligned onto the *Msmg* MmpL3 model to populate both transmembrane and periplasmic binding pockets. The missing residues of each system were modeled based on our previous *Mtb* model [28], utilizing the *Mtb* MmpL3 structure (PDB: 7NVH) [46]. All of the models include a symmetric native mycobacterial inner membrane with the following lipids: 1-tuberculostearoyl-2-palmitoyl-sn-glycero-3-phosphorylethanolamine (TPPE), cardiolipin (10,30-bis[1-oleoyl-2-palmitoleoyl-sn-glycero-3-phospho]-glycerol), 1-palmitoyl-2-oleoyl-sn-glycero-3-phosphatidylinositol (POPI), and four varieties of acylated phosphatidylinositol mannosides, AcPIM_2_, Ac_2_PIM_2_, AcPIM_6_, and Ac_2_PIM_6_. The total number of each membrane component was set to TPPE, 88; cardiolipin, 118; POPI, 6; AcPIM_2_, 35; Ac_2_PIM_2_, 35; AcPIM_6_, 17; and Ac2PIM_6_, 17. All systems were solvated with TIP3P water above and below the protein [52]. Sodium (Na^+^) and chloride (Cl^–^) ions were added to the bulk water to achieve a concentration of 150 mM. The final size was approximately 250,000 atoms for all systems.

### MD simulation

Simulations were conducted using NAMD2/3 [53,54], with the CHARMM36m force field for proteins and CHARMM36 for lipids [55,56]. Hydrogen mass repartitioning was implemented to facilitate a uniform time step of 4 fs for protonated simulations [57]. The simulations were maintained at a constant temperature of 310 K and a pressure of 1 atm using Langevin dynamics and a Langevin piston, respectively [58–60]. As a starting point, we adopted the equilibrated system from our previous study [28], for which a multi-step relaxation protocol [61] was applied. Harmonic positional restraints with a force constant of ${\text{1 kcal mol}}^{-1}\cdot$Å^–2^ were applied to specific atoms at each step. In the first step, all components except the lipid acyl chains were constrained for 1 ns, allowing them to become fluid. Following this, we released all system components except for the protein for 10 ns. Next, the protein side chains were released, and an additional 10 ns was run. The entire system was released for a 100-ns equilibration. After ensuring that the protein and membrane were relaxed, we generated four protonation-state systems: the non-protonated system (NP), the Asp256-protonated system (D256(prot.)), the Asp645-protonated system (D645(prot.)), and the dual-protonated system in which both Asp256 and Asp645 were protonated (both). The D256(prot.) and D645(prot.) systems were generated by protonating Asp256 or Asp645, respectively, while NP retained the standard unprotonated Asp256 and Asp645 states. D645(prot.), D256(prot.) and NP systems were simulated for 2 μs with five replicas per system, whereas the dual-protonated system was simulated for 2 μs with three replicas.

To test whether local hydrogen-bonding rearrangements can reproduce the conformational effects observed in the protonated systems, we performed additional restrained MD simulations starting from the NP system. For the Res-D256H simulation, harmonic distance restraints were applied to the Asp256-Ala682 and Asp645-Ser293 hydrogen-bond distances, with a target distance of 3 Å and a force constant of ${\text{5 kcal mol}}^{-1}\cdot$Å^–2^. For the Res-D645H simulation, a harmonic distance restraint was applied to the Asp645-Tyr257 hydrogen-bond distance using the same target distance and force constant. Each restrained simulation was carried out for 400 ns. Subsequently, TMM was introduced into either the periplasmic or the transmembrane binding pocket for the NP, D256(prot.), and D645(prot.) systems, and these simulations were carried out for 1 μs, again with three replicas per system. In total, 54 μs of unrestrained simulation data, plus 0.8 μs of restrained simulation data, were generated (S1 Table).

## Results

### Protonated states influence the dyad conformation and water accessibility

To investigate how protonation influences the Asp-Tyr dyads, we conducted MD simulations of MmpL3 under three distinct protonation conditions: the unprotonated (NP) state, the Asp256-protonated (D256(prot.)) state, and the Asp645-protonated (D645(prot.)) state (Fig 1A). These fixed-protonation simulations were designed to model putative proton-transfer intermediates and to isolate the structural consequences of protonation at each conserved aspartate. This treatment is consistent with recent QM/MM simulations of MmpL3 proton transfer, which showed that protonation of the conserved aspartates can occur rapidly through transient water chains in the upper chamber and that subsequent deprotonation from the proton gate requires additional proton-transfer steps through the lower chamber [62]. As an additional completeness test, we also simulated a dual-protonated state in which both Asp256 and Asp645 were protonated. The dual-protonated system sampled conformations that largely overlapped with the NP, D256(prot.), and D645(prot.) ensembles in the dyad position clouds and in the transmembrane- and periplasmic-pocket distance distributions (S8 Fig), indicating that simultaneous protonation does not introduce an additional distinct conformational state beyond those characterized by the singly protonated systems. The NP, D256(prot.), and D645(prot.) conditions were each simulated in five replicas, yielding a cumulative sampling time of approximately 10 μs per condition. Root-mean-square deviation (RMSD) analyses confirmed that all systems remained structurally stable throughout the simulations, with no substantial global conformational rearrangements observed (S1 Fig).

A detailed examination of the Asp-Tyr dyads (Asp256, Asp645, Tyr257 and Tyr646) revealed notable local conformational changes (Fig 1C and 1D). In the D256(prot.) system, Asp256 frequently adopted an outward-facing orientation in which its side chain points away from the dyad center, effectively “opening” the dyad. This conformation is postulated to facilitate proton exchange by transiently exposing the dyad to the surrounding solvent. In contrast, Asp645 in the D645(prot.) system remained more constrained, predominantly maintaining an inward-facing orientation that stabilized the dyad in a “closed” state. These distinct conformational preferences were accompanied by changes in the surrounding hydrogen-bond network (Fig 1B and 1C). In the D256(prot.) state, the protonated Asp256 frequently forms a H-bond with the backbone of Ala682, an interaction unique to this state. This contact pulls the Asp256 side chain outward, promoting the open conformation. Conversely, in the D645(prot.) state, Asp645 forms significantly fewer H-bonds with Ser293, while also producing a greater but highly variable propensity for interaction with Tyr257. Tyr257 may therefore contribute to the local conformational preference of the dyad by modulating Asp side-chain orientation and packing. Notably, the residue Ser293 (corresponding to Ser288 in *Mtb*) has been previously identified as a site where mutation (S288T) confers broad-spectrum resistance to MmpL3 inhibitors [63,64]. Our observations suggest that Ser293 may contribute to the balance between open and closed dyad conformations and thereby influence access to this region during protonation-coupled conformational cycling.

To further analyze dyad dynamics under different protonation states, we analyzed the distance distributions between the side chains of the dyad residues as well as the projected cross-sectional area of the binding site in the *x*-*y* plane (S2 Fig). The NP system exhibited the greatest conformational flexibility, as evidenced by broader distributions in both inter-residue distances and projected area defined by the four dyad residues (31.9±4.7 Å^2^). After protonating the selected aspartic acid, the D256(prot.) and D645(prot.) systems displayed more restricted dyad movements, suggesting that the local conformational change induced by protonation imposes constraints that effectively “lock” the dyad into a preferred state. Specifically, protonation at Asp256 increased the average distances between both Asp645–Asp256 and Tyr646–Asp256 pairs, effectively expanding the local cavity near the dyad (42.0±2 Å^2^) (S2 Fig). Conversely, protonation at Asp645 reduced these distances, tightening the projected area defined by dyad residues (29.2±2.1 Å^2^). The difference was significant for D256(prot.) relative to D645(prot.) (Welch’s *t*-test on per-replica means, *n* = 5, with *p* values adjusted within each panel by the Benjamini–Hochberg (BH) procedure to control the false discovery rate; $p=2.9\times 10^{-4}$). Consistent with this expansion, the Asp645–Asp256 and Tyr646–Asp256 distances were each approximately 1 Å larger in D256(prot.) than in D645(prot.) (both BH-adjusted *p* = 0.010). These findings suggest that protonation at different sites exerts opposing effects on the dyad’s structural dynamics, with D256(prot.) promoting a more open and solvent-accessible configuration and D645(prot.) stabilizing a more compact and enclosed one.

The observed conformational changes were further correlated with alterations in local hydration patterns near the dyads. Water-density maps revealed enhanced hydration on the TM4 side of the dyad in the D256(prot.) system, including a high-density region between TM4 and TM6 (Fig 2B). Residue-level water-contact analysis, however, showed a heterogeneous response along TM4. Several residues between 248 and 254 showed a modest trend toward increased water contact, whereas residue 259 exhibited a pronounced decrease in the D256(prot.) system (Figs 2D and S3). In contrast, protonation at Asp645 produced a more consistent reduction in hydration along TM10, largest at residue 648 (0.30 versus 0.91 in NP; raw *p* = 0.0030, BH-adjusted *p* = 0.084), consistent with the reduced water density observed on this side of the dyad (Figs 2C, 2E, and S3). Together, these results indicate that protonation at Asp256 and Asp645 produces distinct and spatially heterogeneous changes in the local hydration landscape, with Asp645 protonation favoring a less hydrated TM10-side configuration.

**Fig 2:**
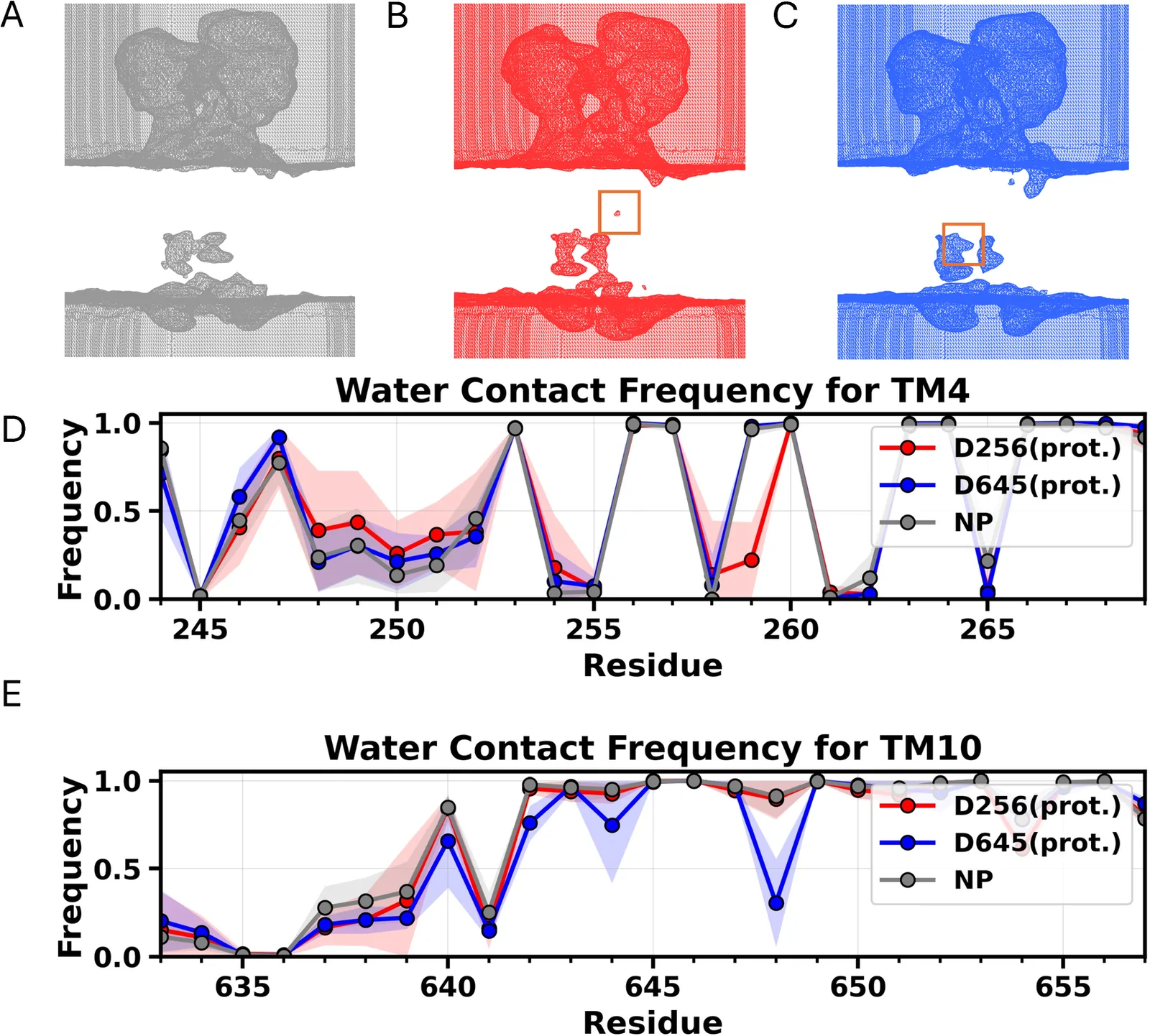
Water accessibility near the dyads. **(A-C)** Water density map for NP (grey), D256(prot.) (red), and D645(prot.) (blue), generated by combining trajectories from all five replicas for each protonation state and shown at an isosurface value of 0.17. The orange boxes in panels B and C highlight the area of differential density, which is also identified by the water contact frequency plot. Protein orientation and detailed, zoomed-in views of these regions are shown in S3 Fig. **(D)** Average water contact frequency for residues located along TM4. **(E)** Average water contact frequency for residues located along TM10. Solid lines with markers show the mean across the five replicas of each protonation state, and the shaded bands span mean ± 1 SD across replicas; the replica-resolved profiles are shown in S3 Fig.

Collectively, these findings support a model in which protonation at distinct sites (Asp256 vs. Asp645) modulates the dyads’ conformations and reshapes the local hydration landscape, thereby potentially influencing the efficiency of proton transfer through MmpL3.

### Protonated states lead to conformational change in both periplasmic and transmembrane domains

To explore the dynamic and conformational changes in the periplasmic domain (PD) of MmpL3, we conducted a focused analysis of this region. Both protonated systems sampled a broader range of PD conformations across replicas than the NP system, which showed highly reproducible PD positioning relative to the transmembrane domain (S1 Fig), suggesting that protonation increases replica-to-replica conformational variability of the PD.

The conformational changes in the PD are particularly relevant at two key functional sites. The periplasmic N-terminal (PN) and C-terminal (PC) interface contains the periplasmic binding pocket [29], whereas the PD–TM interface forms the transmembrane binding pocket where TMM binds spontaneously [28]. Because MmpL3 mediates the transfer of TMM from the transmembrane binding pocket to the periplasmic binding pocket, characterizing conformational changes in the PD is critical for understanding the transport mechanism. To investigate changes near the transmembrane binding pocket (formed by TM7 and TM8 at the PD-TM interface), we analyzed distance matrices between residues 550–565 (TM8) and 412–427 (TM7) (Figs 3A and S5). Comparison of the distance-difference matrices between the D256(prot.)–NP and D645(prot.)–NP systems revealed a consistent pattern characterized by increased distances in the bottom-right regions and decreased distances in the top-left regions. Mapping these changes onto the MmpL3 structure highlights distinct spatial rearrangements (Fig 3B). Specifically, the upper regions of the binding pocket showed reduced distances, suggesting tighter packing, while the lower regions exhibited increased separation. These protonation-associated rearrangements reshape the geometry of the transmembrane pocket and may influence substrate positioning during transport.

**Fig 3:**
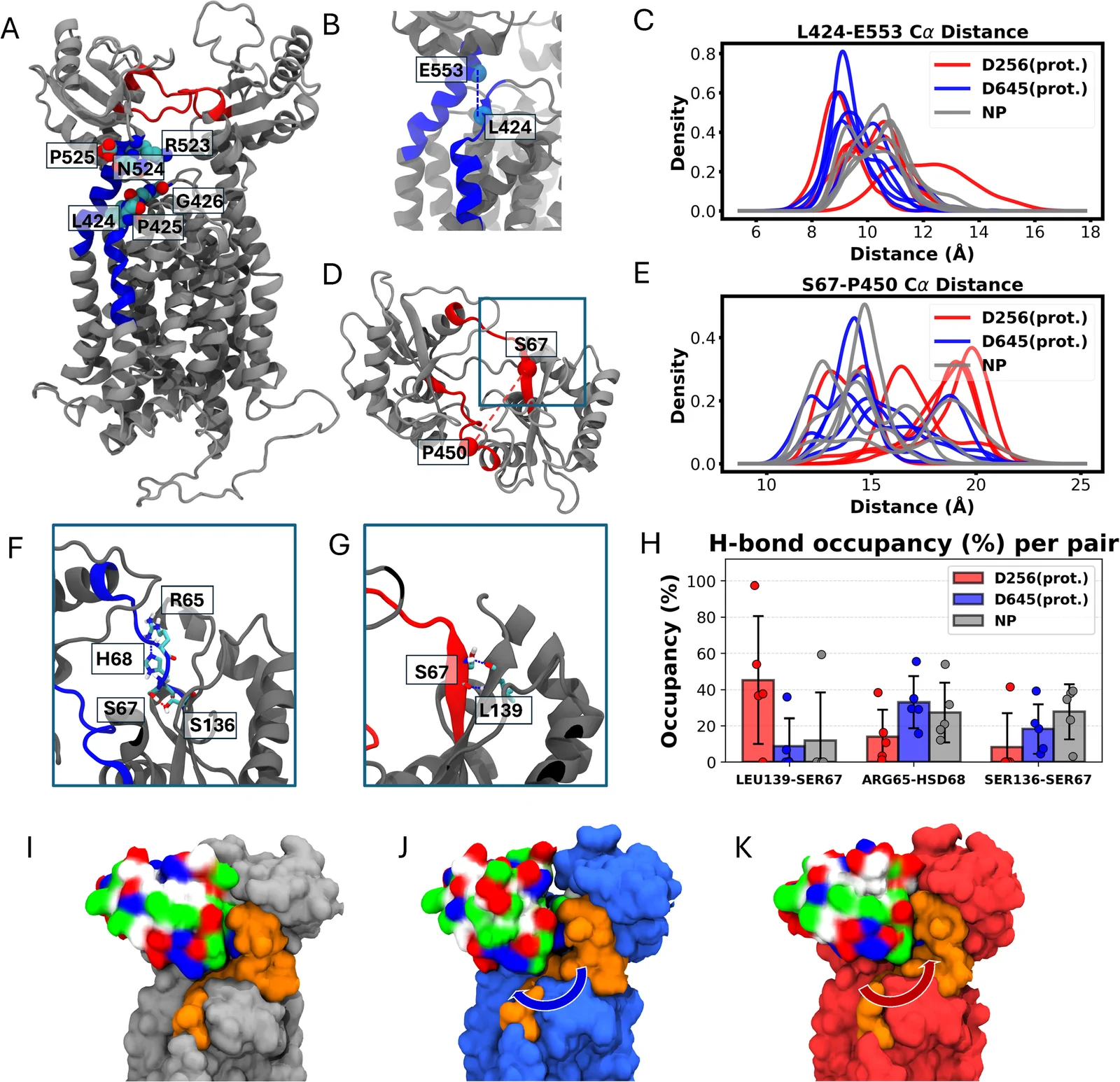
Protonation-dependent conformational changes in the substrate-binding pockets of *Msmg* MmpL3. **(A)** Overview of MmpL3 highlighting the locations of the transmembrane (TM, blue) and periplasmic (red) substrate-binding pockets. Gating residues lining the TM binding pocket are shown as spheres, with oxygen colored red, nitrogen blue, and carbon cyan. **(B-C)** Zoomed-in view and Cα-Cα distance distribution for the TM pocket entrance, defined by Leu424 and Glu553, in NP (grey), D256(prot.) (red), and D645(prot.) (blue). **(D-E)** Periplasmic-side view and Cα-Cα distance distribution for the periplasmic pocket opening, defined by Ser67 and Pro450. **(F-G)** Representative hydrogen-bond networks near the periplasmic pocket in the D645(prot.) and D256(prot.) systems. **(H)** Hydrogen-bond occupancies for representative periplasmic-pocket interactions; bars show mean occupancies across five replicas, points show individual replicas, and pairwise Welch’s *t*-test *p* values are reported in S1 Fig. **(I-K)** Surface representations viewed from the periplasmic side for the NP (I, grey), D645(prot.) (J, blue), and D256(prot.) (K, red) systems. Residues 451-550 are colored by residue type: hydrophobic, white; hydrophilic, green; negatively charged, red; and positively charged, blue. Residues 418-450 are shown in orange. The curved arrows indicate *t*he relative displacement of the lower portion of residues 451-550 with respect to residues 418-450, illustrating protonation-dependent rotation near the interface between the periplasmic domain and the transmembrane domain.

To quantify pocket width, we tracked the distance between the Cα atoms of Glu553 and Leu424, two residues lining the periplasm-facing entry of the transmembrane binding pocket at the PD–TM interface (Fig 3C). The D645(prot.) system sampled a narrower pocket than NP (9.7±0.4 versus 10.4±0.3 Å, BH-adjusted *p* = 0.044), with four of five replicas falling below the entire range sampled by NP. In contrast, D256(prot.) had a similar mean pocket width to NP (10.4±1.2 Å) but showed substantially greater replica-to-replica variability. Four D256(prot.) replicas sampled predominantly closed-like distributions with mean distances of approximately 9.0–10.4 Å, whereas one replica sampled a distinctly wider distribution peaking near 12 Å. These results suggest that Asp645 protonation biases the transmembrane-pocket entrance toward a narrower conformation, whereas Asp256 protonation increased conformational heterogeneity, with one replica sampling a markedly more open pocket conformation.

Moreover, trajectory inspection revealed coordinated rigid-body movements between the PN and PC domains. Quantitative analysis of domain rotations revealed protonation-dependent differences primarily along the *x*-axis (S4 Fig). The PC domain showed a larger mean *x*-axis rotation in D256(prot.) than in the D645(prot.) or NP systems. A closer examination revealed that the lower region of the PC domain tended to rotate clockwise in the D645(prot.) system (Fig 3J) and counter-clockwise in the D256(prot.) system (Fig 3K), when viewed from the periplasm. Given the location of this region near the PD-TM interface, these movements represent a plausible conformational change that could be relevant for substrate translocation. To quantify this, we measured the center-of-mass distance between residue groups 523–525 and 423–425, which define the narrowest portion of the TMM transport channel [29] (Fig 3A). The mean gate separation was 14.1±0.9 Å in D256(prot.), 13.5±0.6 Å in NP, and 13.2±0.5 Å in D645(prot.) (S6 Fig). D256(prot.) therefore showed a tendency toward a wider gate than D645(prot.). These observations are consistent with a tendency for D256(prot.) to sample more open configurations at this interface, whereas D645(prot.) favors more compact configurations.

We performed a parallel analysis at the periplasmic pocket by computing distance matrices between residues 60–70 and residues 448–458 (Figs 3A and S5). The contact map reveals notable differences around residues 65–67 in the D256(prot.) system, which correspond to an opening of the periplasmic pocket (Fig 3D). To further quantify this change, we measured the Cα–Cα distance between Ser67 and Pro450 (Fig 3E). The D256(prot.) system tended to sample larger distances (17.4±2.1 Å) than either D645(prot.) (15.1±1.3 Å, BH-adjusted *p* = 0.112; raw *p* = 0.075) or NP (15.0±1.3 Å, BH-adjusted *p* = 0.112; raw *p* = 0.074). Concurrently, the hydrogen-bond network near the periplasmic pocket also changed (Fig 3F–3H). The Leu139–Ser67 interaction was most frequently observed in D256(prot.) (45±35%), while it was sampled sporadically in individual NP and D645(prot.) replicas. Relative to D256(prot.), D645(prot.) showed higher occupancies of the Arg65–His68 and Ser67–Ser136 interactions. Together, these results indicate that protonation shifts the balance among alternative hydrogen-bonding arrangements near the periplasmic pocket. Supporting the functional relevance of this region, Ser66 in *Mtb*, which corresponds to Ser67 in our system, has been shown experimentally to influence proton transfer and growth in *Msmg* [30], and the S66A mutation induces conformational changes in the same TMM-binding loop. Overall, these results indicate subtle protonation-dependent rearrangements, with the D256(prot.) system tending to favor more open configurations, whereas D645(prot.) favors more compact configurations.

To further test whether local dyad rearrangements can allosterically influence the substrate-binding pockets, we performed additional restrained simulations in which selected dyad hydrogen-bond distances were restrained to mimic the local configurations observed in the D256(prot.) and D645(prot.) systems. These restrained simulations partially reproduced the corresponding shifts in the periplasmic- and transmembrane-pocket distance distributions (S9 Fig), consistent with the idea that local conformational changes at the Asp–Tyr dyads contribute to allosteric remodeling of the substrate-binding pockets.

Using dynamic network analysis [65], we identified two candidate allosteric communication pathways connecting the dyads to the substrate-binding pockets (S5 Fig). The pathway originating from Asp256 primarily connects to the periplasmic binding pocket through TM2, while the pathway from Asp645 links to the transmembrane binding pocket through TM8. These pathways provide plausible routes by which local protonation-dependent rearrangements at the dyads can propagate to distant substrate-binding regions, supporting allosteric communication between protonation state and sites involved in substrate transport.

### Protonated aspartates allosterically couple with substrate binding at both binding pockets

Given the observation that protonation at the Asp–Tyr dyads influences conformations near both TMM-binding sites, we performed additional MD simulations to further clarify how dyad protonation affects MmpL3–TMM interactions. Specifically, we constructed NP, D256(prot.), and D645(prot.) systems, each with TMM placed either in the transmembrane binding pocket or in the periplasmic binding pocket.

Across all conditions, TMM remained stably bound within the transmembrane binding pocket throughout the simulation period. In all three systems, TMM remained within the same transmembrane binding pocket and formed recurrent hydrogen-bonding interactions with a common set of residues, including Leu424, Gln554, and His558. However, the hydrogen-bonding pattern of the TMM headgroup within this shared pocket showed protonation-associated trends. In the non-protonated system, TMM more frequently formed hydrogen bonds with residues at the acidic end of the pocket, farther from the central transport channel, including Asp525, Lys528, Asp531, Asp562, and Pro565 (Figs 4A and S7C). TMM formed at least one hydrogen bond with this region in 28% of NP frames, compared with only 6% in D256(prot.) and 9% in D645(prot.). The same pattern was observed across individual replicas, with occupancies of 20–33% in the three NP replicas but no more than 11% in any of the six protonated replicas (BH-adjusted *p* = 0.083; two-sided Welch’s *t*-test on per-replica occupancies).

**Fig 4:**
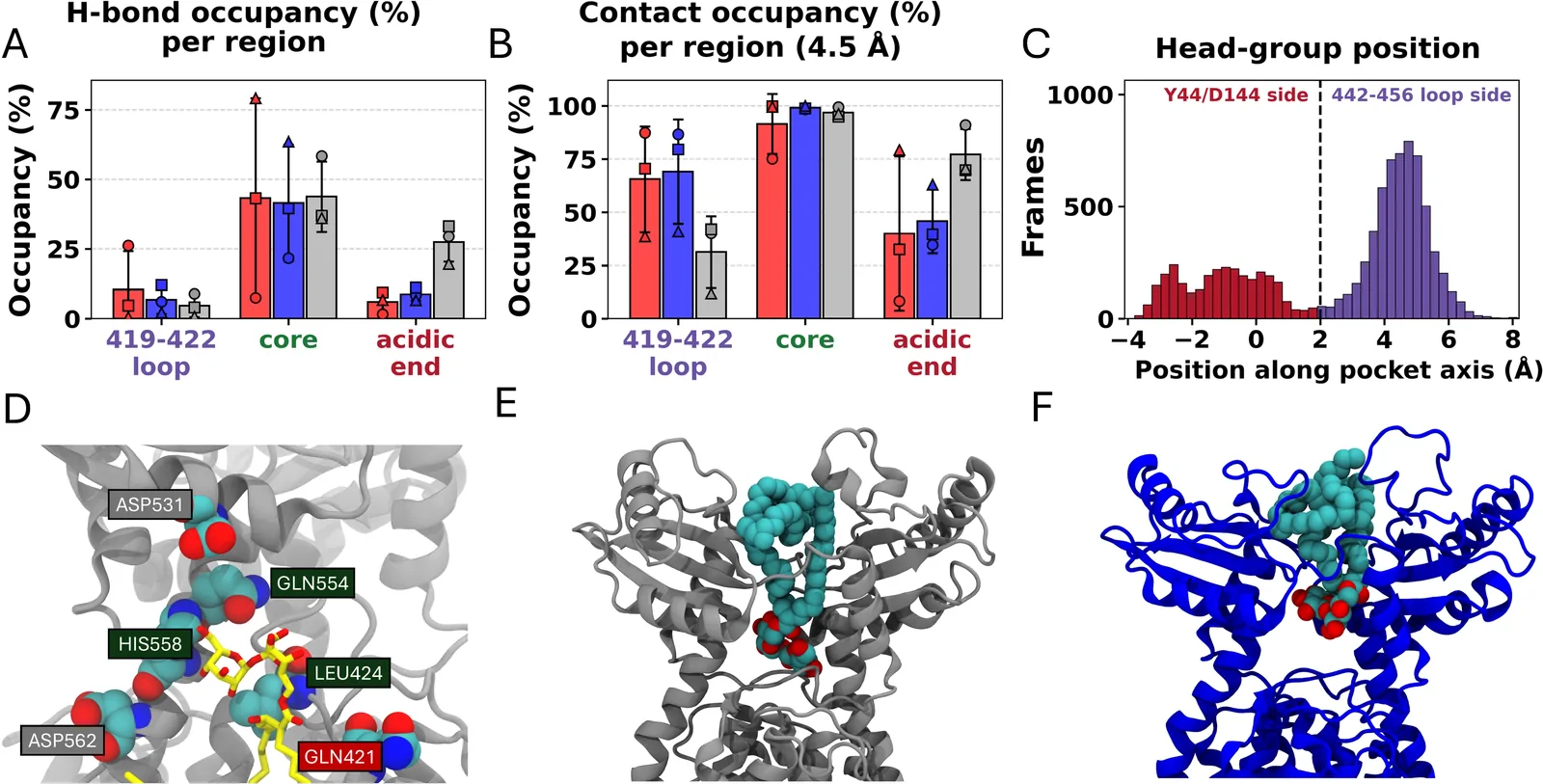
TMM binding patterns across Asp dyad protonation states. **(A)** Hydrogen-bond occupancy between TMM and regions of the transmembrane binding pocket, and (B) contact occupancy for the same three regions using a 4.5 Å distance cutoff. Residues comprising each region are listed in S7 Fig. Bars show means across three replicas, error bars indicate standard deviations, and symbols show individual replicas. **(C)** Distribution of the TMM headgroup position along the internal axis of the periplasmic pocket, pooled across all nine trajectories. The bimodal distribution reveals two recurrent binding sub-sites: a Tyr44/Asp144-side sub-site and a 442–456 loop-side sub-site. The dashed line at 2.0 Å indicates the boundary used for sub-site assignment. BH-adjusted *p* values for the pooled protonated versus NP comparison are reported in S7C Fig. **(D)** Representative TMM-bound structure in the transmembrane pocket, with residues colored by preferential interaction across protonation states. **(E,F)** Representative TMM configurations in the two periplasmic-pocket sub-sites: (E) the NP system, in which TMM occupies the Tyr44/Asp144-side sub-site, and (F) the D645(prot.) system, in which TMM occupies the 442–456 loop-side sub-site. Additional residue-level interactions, sub-site depth, and replica assignments are shown in S7 Fig and S2 Table.

Consistent with this pattern, the protonated systems showed a trend toward positioning the TMM headgroup closer to the Leu419–Leu422 side of the pocket. Relative to NP, the protonated systems showed an average displacement of approximately 2.3 Å along the pocket axis. Contact with the Leu419–Leu422 loop tended to be higher in the protonated systems, increasing from 31% in NP to 66–69% (BH-adjusted *p* = 0.059; two-sided Welch’s *t*-test on per-replica occupancies), whereas contact with the acidic end tended to be lower, decreasing from 77% in NP to 40–46% in the protonated systems (BH-adjusted *p* = 0.059; two-sided Welch’s *t*-test on per-replica occupancies; Figs 4B and S7C).

One D256(prot.) replica additionally sampled a configuration in which TMM formed a hydrogen bond with Gln421 (S7B Fig). This interaction reached 46% contact occupancy and 21% H-bond occupancy in that trajectory but was rare in the other eight trajectories. Overall, the simulations suggest a trend in the protonated systems toward reduced sampling of the NP-favored acidic end of the pocket and toward positions closer to the central channel.

At the periplasmic site, TMM remained bound throughout all simulations. The core of the pocket (Ser67, Gly170, Leu171, Ile427, Glu429, and Thr549) was contacted in nearly every frame, and the fraction of frames containing at least one hydrogen bond to this region was similar among D256(prot.), D645(prot.), and NP (93%, 88%, and 88%, respectively). The TMM headgroup nevertheless sampled different positions within the pocket. Projection along the internal pocket axis revealed that this variation reflects two recurrent binding sub-sites rather than a continuously sampled displacement (Fig 4C). These correspond to a 442–456 loop-side sub-site and a Tyr44/Asp144-side sub-site, separated by a sparsely populated region along the pocket axis (S7G–S7J Fig).

The two sub-sites were associated with distinct hydrogen-bonding patterns. In the 442–456 loop-side sub-site, the trehalose headgroup preferentially interacted with residues including Glu455, Glu429, Arg453, Gln442, Thr454, and Thr66, whereas the Tyr44/Asp144-side sub-site was characterized by stronger interactions with Ser67, Leu171, and Tyr44 (S7D and S7F Fig). The two sub-sites also differed in their depth within the pocket: the 442–456 loop-side configuration positioned the headgroup closer to the periplasm than the Tyr44/Asp144-side configuration (26.7 ± 1.5 Å versus 22.4 ± 1.6 Å from the protein center of mass, *p* = 0.024; Figs 4E, 4F, and S7E). Thus, movement between the two sub-sites combines a lateral repositioning within the pocket with displacement toward the periplasm.

Individual replicas showed little exchange between the two sub-sites on the simulated timescale. Eight of the nine trajectories spent either more than 93% or less than 1% of their frames in the 442–456 loop-side sub-site (S2 Table). All three D645(prot.) replicas predominantly sampled the loop-side sub-site, compared with two of three D256(prot.) replicas and one of three NP replicas. Because individual trajectories largely remained within a single sub-site, these counts describe preferential state sampling rather than converged equilibrium populations and are therefore reported descriptively. The stronger sampling of the loop-side sub-site in the protonated systems, particularly D645(prot.), is consistent with their more periplasmic TMM headgroup positions. A comparable repositioning of TMM in the periplasmic pocket has been reported for the *Mtb* H68A mutant, which is allosterically coupled to proton transport and was proposed to create a barrier to substrate escape [30].

Together, these results suggest that dyad protonation is associated with preferential sampling of distinct TMM binding sub-sites within the periplasmic pocket, with the 442–456 loop-side configuration positioned closer to the periplasm and sampled most consistently in D645(prot.).

## Discussion

This work provides a mechanistic view of how protonation at the central Asp–Tyr dyads modulates the structure, hydration, and substrate interactions of MmpL3. By comparing three systems that differ only in the protonation state of the dyads, we identify a coherent pattern linking local chemical changes at the dyads to long-range motions of the periplasmic domain and to coupled changes in the two substrate-binding pockets.

At the level of the dyads, protonation at Asp256 favors an outward orientation of the Asp256 side chain and reorganizes local hydration on the TM4 side, whereas protonation at Asp645 stabilizes an inward orientation and reduces hydration on the TM10 side. These distinct conformational preferences are accompanied by different hydrogen-bonding networks. In particular, backbone contacts between protonated Asp256 and Ala682 are associated with the open configuration, while Asp645 protonation shows a higher but variable propensity for interaction with Tyr257, which may contribute to a more closed arrangement. Notably, the sensitivity of contacts involving Ser293 (corresponding to Ser288 in *Mtb*) has previously been linked to inhibitor resistance, supporting a role for this residue in regulating access to the dyad region and in modulating the balance between open and closed substrate-access states.

Furthermore, protonation-dependent changes at the dyads propagate to the periplasmic domains and to both substrate-binding pockets. Distance-based metrics indicate that protonation alters the geometry of the cavity formed by the transmembrane helices, with distinct effects depending on the protonation site. Specifically, the Cα–Cα distance between Leu424 on TM7 and Glu553 on TM8 shows that D645(prot.) favors a narrower pocket entrance, whereas D256(prot.) shows greater conformational variability and occasionally samples more open states. Consistent with this trend, rotation of the periplasmic subdomain and gating motions at the PD–TM interface, defined by residue groups 523–525 and 423–425, follow the same ordering across protonation states. At the periplasmic binding pocket formed at the PN–PC interface, the separation between the two loop regions, measured by the distance between Ser67 and Pro450, tended to be larger in D256(prot.) than in D645(prot.) or NP. Together, these observations support a mechanical linkage in which dyad protonation biases periplasmic domain motions that modulate gating at the transmembrane pocket and reshape the periplasmic pocket.

The hydration analysis provides an important connection between local chemistry at the dyads and larger-scale conformational behavior. Proton transfer requires transient solvent connectivity, and protonation-dependent reorganization of hydration on the TM4 side in the D256(prot.) state, including enhanced local water density near the TM4–TM6 region, offers a plausible route for charge stabilization and for subsequent protonation steps. In contrast, the reduced hydration on the TM10 side in the D645(prot.) state is consistent with a configuration that is less permissive to further proton exchange. Based on these observations, we favor a sequential model in which protonation at Asp256 occurs first, reorganizing local hydration and shifting the conformational ensemble toward states that may facilitate subsequent protonation at Asp645. Subsequent protonation at Asp645 then shifts the conformational ensemble toward a more compact and less hydrated state, which is compatible with closure of the pathway and forward progression of the transport cycle.

Simulations with TMM showed trends consistent with this model. In the transmembrane pocket, the protonated systems tended to shift the TMM interaction pattern away from the NP-favored acidic end toward the central transport channel. In one D256(prot.) replica, this repositioning was accompanied by an additional interaction with Gln421. In the periplasmic pocket, the 442–456 loop-side configuration was sampled by more protonated trajectories, with all three D645(prot.) replicas occupying this sub-site. These protonation-dependent differences in TMM positioning are consistent with the accompanying periplasmic-domain motions and pocket-gating rearrangements.

Together, the data support an alternating-access model [66] in which changes in protonation bias the conformational landscape of MmpL3. In the NP state, the protein samples both open-like and closed-like local substates at the dyads. Protonation at Asp256 favors an open and hydrated dyad configuration, accompanied by an increased separation between the dyads and a tendency toward expansion of the periplasmic pocket. In contrast, protonation at Asp645 stabilizes a compact dyad arrangement, reduces this separation, and promotes a recessed conformation in the periplasmic domain. Following substrate release and deprotonation, the system may return toward the NP ensemble, thereby enabling another round of transport. On the basis of these observations, we propose a refined model for MmpL3’s transport cycle: i) Unprotonated state: MmpL3 initially binds TMM in the transmembrane pocket. ii) Protonation at Asp256: promotes an open and hydrated conformation and repositions TMM toward the central channel, priming the system for substrate movement. iii) Protonation at Asp645: favors a more compact conformation and TMM configurations shifted toward the periplasmic side, consistent with forward progression of the transport cycle (Fig 5).

**Fig 5:**
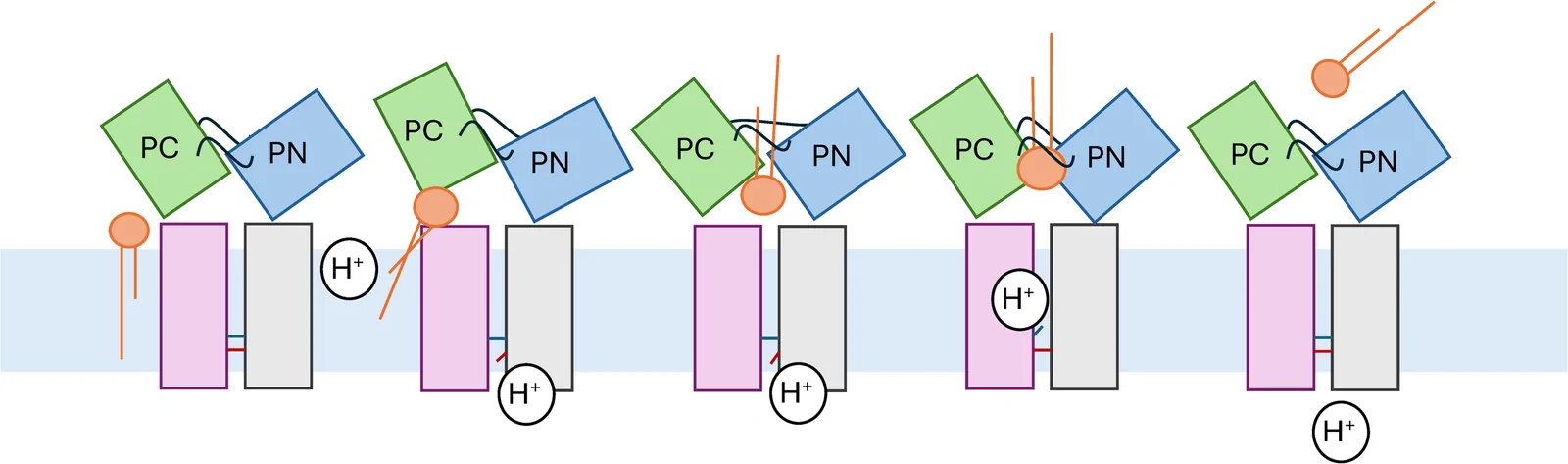
Proposed mechanism of MmpL3 lipid transport. TMM (orange) is transferred from the transmembrane binding pocket to the periplasmic side, from left to right. The green block represents the PC domain, the blue block represents the PN domain, and the two connecting loops form the periplasmic binding pocket for TMM. The grey block represents TM1 to TM6, with Asp256 and Tyr257 at the center. The purple block represents TM7 to TM12, with Asp645 and Tyr646 at the center. Red lines represent interactions between Asp256 and Tyr646. Blue lines represent interactions between Asp645 and Tyr257.

A limitation of the present study is the sampling accessible to conventional fixed-protonation MD. Although our multi-replica microsecond simulations reveal consistent protonation-dependent shifts in dyad structure, hydration, domain motions, and TMM binding, they do not fully define the free-energy landscape or transition barriers between states. The exact H^+^/TMM stoichiometry of MmpL3 also remains unknown, and resolving this question will require experiments that directly couple measurements of proton flux with TMM transport. Future enhanced-sampling and free-energy approaches could more rigorously quantify the stability of the observed open and closed conformations and the pathways connecting them.

Another potential future direction is to clarify whether the conserved Tyr residues play a more direct role in proton transfer. Our simulations suggest that Tyr257 and Tyr646 help shape the local hydrogen-bonding environment and stabilize distinct Asp256/Asp645 conformations, but fixed-protonation classical MD cannot determine whether these Tyr residues transiently donate or accept protons. Recent QM/MM simulations of MmpL3 have primarily implicated water-mediated proton transfer to and from the conserved Asp residues rather than direct protonation of Tyr itself [62]. Future QM/MM, constant-pH MD, or related reactive simulations will be needed to test whether Tyr257 and Tyr646 act solely as structural/electrostatic modulators of the dyads or also participate directly in proton exchange.

## Supporting information

S1 Fig Stability of MmpL3 simulations and protonation-dependent H-bond networks. (A) RMSD time series for the NP, D256(prot.), and D645(prot.) systems across five replicas. Hydrogens were excluded from the RMSD calculations. The top-left panel shows the global protein RMSD, the top-right panel shows the periplasmic domain (PD) RMSD, the bottom-left panel shows the global protein RMSD after excluding the flexible loop region (residues 341–399), and the bottom-right panel shows the transmembrane domain (TMD) RMSD. **(B)** H-bond occupancies for dyad-proximal interactions involving Ser293-Asp645, Asp645-Tyr257, and Asp256-Ala682. Bars show the mean occupancy across five replicas, individual points indicate replica values, and error bars represent standard deviations. Pairwise BH-adjusted *p* values are shown above the bars. **(C)** H-bond occupancies for periplasmic-pocket interactions involving Leu139-Ser67, Arg65-His68, and Ser136-Ser67. Bars show the mean occupancy across five replicas, individual points indicate replica values, and error bars represent standard deviations. Pairwise BH-adjusted *p* values are shown above the bars.
(TIFF)

S2 Fig Protonation-dependent rearrangement of the Asp–Tyr dyads in MmpL3. Top: Time evolution of the enclosed polygon area defined by the OD1/OH atoms of the Asp–Tyr dyads for the NP, D256(prot.), and D645(prot.) systems. Smoothed trajectories are shown using a 10-frame rolling average for five independent replicas of each system. Middle and bottom: For each residue pair, left panels show representative structural snapshots highlighting the two residues used in the analysis, and right panels show kernel density estimates of inter-residue distance distributions for NP (grey), D645(prot.) (blue), and D256(prot.) (red). The analyzed residue pairs include Asp645–Asp256, Asp645–Tyr257, Tyr646–Asp256, and Tyr646–Tyr257.
(TIFF)

S3 Fig Replica-resolved water contact frequency across transmembrane helices. (A) Normalized water contact frequency for residues located along TM1-TM12 in the NP, D256(prot.), and D645(prot.) systems. Thin traces show individual replicas, with line style denoting replica identity, and thick traces show the mean profile for each protonation state. (B, C) Protein orientation with the corresponding water density maps for the D256(prot.) (B, red) and D645(prot.) (C, blue) systems. The zoomed-in panels show the highlighted regions with altered hydration. Labeled residues indicate positions with altered water contact frequencies.
(TIFF)

S4 Fig Rotation angle distributions of the periplasmic N-terminal (PN, residues 38–180) and C-terminal (PC, residues 425–528) domains relative to the reference structure. The left panel shows the MmpL3 structure with the coordinate axes indicated. Top row: kernel density estimates of PN domain rotations about the X, Y, and Z axes for the NP, D256(prot.), and D645(prot.) systems. Bottom row: corresponding rotation angle distributions for the PC domain.
(TIFF)

S5 Fig Protonation-dependent structural changes and allosteric pathways connecting the Asp–Tyr dyads to substrate-binding pockets. (A) Representative structural view and residue-pair distance difference maps for the periplasmic binding pocket, calculated between residues 60–70 and 448–458. Difference maps are shown for D256(prot.)–NP and D645(prot.)–NP. Positive values indicate increased distances relative to the non-protonated system, whereas negative values indicate decreased distances. (B) Representative structural view and residue-pair distance difference maps for the transmembrane binding pocket, calculated between residues 550–565 and 412–427. (C, D) Dynamic network analysis mapped onto the full MmpL3 structure. Residues along the identified allosteric pathways are shown as cyan spheres, with the associated helices highlighted in blue and the connected pocket regions highlighted in yellow. The pathway associated with TM8 links Asp645 to the transmembrane binding pocket (C), whereas the pathway associated with TM2 links Asp256 to the periplasmic binding pocket (D). Together, these analyses show that protonation-dependent local changes at the dyads are connected to distal substrate-binding pockets through defined structural pathways.
(TIFF)

S6 FigInter-residue distance distributions at the periplasmic gating region of MmpL3.The left panel shows the MmpL3 structure with the gating residues highlighted as spheres. Residues 423–425 on one side of the gate and residues 523–525 on the opposing side define the narrow region of the proposed TMM transport pathway. The right panels show kernel density estimates of pairwise distances between opposing gate residues for the NP (grey), D256(prot.) (red), and D645(prot.) (blue) systems. The top row reports Cα-Cα distances, and the bottom row reports sidechain-sidechain distances for the same residue pairs. Each curve represents one simulation replica. These distributions provide a residue-level view of the gate-width changes summarized by the center-of-mass distance analysis in the main text.(TIFF)

S7 FigTMM binding modes in the transmembrane and periplasmic pockets.(A) Residues defining the Leu419–Leu422 loop, common-pocket core, and acidic-end regions. (B) Representative D256(prot.) configuration showing the replica-specific Gln421 interaction. (C) Region-level contact and hydrogen-bond occupancies in the transmembrane pocket. Bars show means ± SD across replicas; points show individual replicas. Pooled protonated replicas (*n* = 6) were compared with NP (*n* = 3) using two-sided Welch’s *t*-tests with BH correction across regions. (D) Hydrogen-bond occupancies distinguishing the 442–456 loop-side and Tyr44/Asp144-side periplasmic sub-sites, with BH-adjusted *p* values. (E) TMM headgroup depth for the two sub-sites. (F) Frame-pooled hydrogen-bond patterns characteristic of each sub-site. (G–J) Representative structures of the two periplasmic binding states viewed along the membrane normal (G,H) and within the membrane plane (I,J). TMM is shown in teal and characteristic interacting residues are labeled.(TIFF)

S8 FigStructural behavior of the dual-protonated Asp256/Asp645 MmpL3 system.(A) Representative view of the dual-protonated system, in which both Asp256 and Asp645 are protonated. The Asp-Tyr dyad region is shown from the transmembrane domain, with key dyad residues shown in licorice representation. (B) Cα and side-chain oxygen position clouds for the Asp-Tyr dyad residues in the non-protonated (NP, grey), Asp256-protonated (D256(prot.), red), Asp645-protonated (D645(prot.), blue), and dual-protonated (Both, green) systems. Each point represents the projected position from simulation frames after structural alignment. (C) Distance distributions of the Leu424-Glu553 Cα pair, which reports the opening of the transmembrane pocket entrance, using three replicas per condition for a matched comparison. (D) Distance distributions of the Ser67–Pro450 Cα pair, which reports the opening of the periplasmic substrate-binding pocket, using three replicas per condition for a matched comparison.(TIFF)

S9 FigRestrained MD simulations testing the role of local dyad hydrogen-bond geometry.(A,B) Representative overlays of restrained simulations designed to mimic the Asp256-protonated (A, green) or Asp645-protonated (B, green) dyad hydrogen-bond configurations, shown with NP (grey), D256(prot.) (red), and D645(prot.) (blue) structures. Harmonic restraints were applied to selected local hydrogen-bond distances near the dyads. (C,D) Distributions of the Ser67–Pro450 Cα distance (C) and Leu424–Glu553 Cα distance (D), reporting on the periplasmic pocket and transmembrane-pocket entrance, respectively. Dashed curves show restrained simulations, which partially reproduce the protonation-dependent shifts observed in the unrestrained protonated systems.(TIFF)

S1 Table Summary of molecular dynamics simulations performed in this study. (TEX)

S2 Table Assignment of each periplasmic-pocket trajectory to a binding sub-site. For each frame, the TMM headgroup centroid was projected, after pocket alignment, onto an axis from the Tyr44/Asp144-side centroid (residues 40, 41, 43, 44, 144, 175, and 178) to the 442–456 loop-side centroid (residues 442, 445, and 452–456). Projections >2.0 Å were assigned to the 442–456 loop-side sub-site; all others were assigned to the Tyr44/Asp144-side sub-site. Eight of the nine trajectories spent either more than 93% or less than 1% of their frames on the loop side, indicating that transitions between the two sub-sites were rare within individual trajectories.
(TEX)
